# The Prevalence of Underweight, Overweight/Obesity and Their Related Lifestyle Factors in Indonesia, 2014–2015

**DOI:** 10.3934/publichealth.2017.6.633

**Published:** 2017-12-26

**Authors:** Supa Pengpid, Karl Peltzer

**Affiliations:** 1 ASEAN Institute for Health Development, Mahidol University, Salaya, Thailand; 2 Department of Research & Innovation, University of Limpopo, Turfloop, South Africa; 3 HIV/AIDS/STIs and TB Research Programme, Human Sciences Research Council, Private Bag X41, Pretoria 0001, South Africa

**Keywords:** underweight, overweight, obesity, dietary behavior, physical activity, adults, Indonesia

## Abstract

**Objective:**

To quantify the prevalence of underweight and overweight or obesity and its related factors (socio-demographic, health behavior, health status) in a national adult population in Indonesia.

**Material and Methods:**

In a national cross-sectional population-based survey in 2014–15 in Indonesia, 29509 adults (median age 41.0 years, Inter Quartile Range = 22.0, age range of 18–103 years) completed questionnaires and anthropometric measurements. Multinomial logistic regression modelling was used to determine the association between socio-demographic, health behavior and health status factors and underweight and overweight or obesity.

**Results:**

Of total sample (n = 29509), 11.2% measured underweight (13.5% among men and 9.1% among women) (<18.5 kg/m^2^), 39.8% normal weight (48.1% among men and 32.0% among women) and 49.0% had overweight or obesity (≥23 kg/m^2^) (38.3% among men and 58.9% among women); 24.6% of the overall sample had class I obesity (25–29.9 kg/m^2^), and 8.5% had class II obesity (30 or more kg/m^2^). Among different age groups, underweight was the highest among 18–29 year-olds (20.0%) and those 70 years and older (29.8%), while overweight or obesity was the highest in the age group 30 to 59 years (more than 53%). In adjusted multinomial logistic regression, having less education, living in rural areas and not having chronic conditions were associated with underweight status. While better education, higher economic status, urban residency, dietary behavior (infrequent meals, frequent meat, fried snacks and fast food consumption), physical inactivity, not using tobacco, having chronic conditions (diabetes, hypertension, hypercholesterol), and better perceived health and happiness status were associated with overweight or obesity.

**Conclusions:**

A dual burden of both adult underweight and having overweight or obesity was found in Indonesia. Sociodemographic, health risk behavior and health status risk factors were identified, which can guide public health interventions to address both these conditions.

## 1. Introduction

Globally, from 1975 to 2014, the prevalence of underweight decreased from 13.8% to 8.8% in adult men and from 14.6% to 9.7% in adult women, and the prevalence of obesity increased from 3.2% to 10.8% in adult men, and from 6.4% to 14.9% in adult women [1]. In Indonesia in 2013, the national adult (18 years and older) prevalence rate of underweight was 11.1% and the prevalence of overweight or obesity (≥25 kg m^2^) was 30.4% [2],[3]. The prevalence of underweight and overweight among girls (15–18 years) in rural areas was 25% and 10%, respectively, and in urban areas 28% and 10%, respectively, in Indonesia [4]. Similar rates of underweight and overweight or obesity have been reported in countries of the Southeast Asian region. In Bangladesh (35 years and older), 30.4% were underweight and 23.5% had overweight or obesity (≥ 23 kg/m^2^) [5]; in Malaysia (18 years and above), 51.2% had overweight or obesity (≥ 25 kg/m^2^) [6]; in Thailand, 40.9% had overweight or obesity (≥23 kg/m^2^) [7]; and in Vietnam in 2005, 20.9% were underweight and 16.3% had overweight or obesity (≥23 kg/m^2^) [8]. As globally, a decrease in the prevalence of underweight and increase of overweight or obesity over the past 20 years have been reported in Indonesia [9] and other countries in the Southeast Asian region such as Vietnam [8] and Thailand [10].

Undernutrition in adulthood can lead to increased morbidity and mortality and other adverse outcomes [11]. Obesity is a major risk factor for a number of non-communicable diseases, such as cardiovascular disease, hypertension, stroke, diabetes mellitus, and specific forms of cancer leading to increased morbidity and premature mortality [12]. Factors related to adult underweight may include sociodemographic variables, such as being female [8],[13],[14], early adulthood (15–24 years) [14], older adults [5],[14], having lower education [5],[13]–[15], poorer economic background [5],[14],[15], not working [5], being married [15], being a non-Christian [15] and residing in rural areas [8]. For example in India, among “rural young (15–24 years) females from more educated villages had a higher likelihood of underweight relative to those in less educated villages; but for rural mature (>24 years) females the opposite was the case” [15]. Moreover, food insecurity, inadequate food intake, diets low in diversity and with insufficient nutrient density [4] and fear of being fat [16] may be associated with underweight.

Sociodemographic risk factors for having overweight or obesity may include being female [5],[8], middle aged [5],[14], higher education [5], higher economic status [5],[14],[15] and urban residence [5],[8]. Obesogenic behaviors may include the consumption of energy-dense foods high in fat, salt and sugars [18],[19], animal source foods [20], meat [21], fried food [22], inadequate fruit and vegetables consumption [23]. Fish intake [24], low meal frequency [25],[26], physical activity [6],[18],[19] and smoking [27],[28] have been found to be negatively related to obesity. Obesity is linked to a number of chronic conditions such as hypertension, type 2 diabetes mellitus, and dyslipidaemia [3],[29]. Physical and mental health status risk factors for obesity include poor self-reported health status [28], depression [30],[31], lower life satisfaction [32], poor sleep quality [33] and greater happiness [34].

There is a lack of more recent national data on the prevalence of underweight and overweight and obesity and its sociodemographic, behavioral and physical and mental health risk factors in Indonesia. It is important to understand factors driving the dual underweight and obesity burden in Indonesia.

## 2. Materials and Methods

### 2.1. Method

Data were analysed from the “Indonesia Family Life Survey (IFLS-5)”, a continuing demographic and health survey that began in 1993 and had since four rounds of data collection, with the fifth wave (IFLS-5) fielded in late 2014 and completed in 2015 [35]. The community surveys collected data on household, individual, and community level using a multistage stratified sampling [35]. The sampling frame of the first survey in 1993 was based on households from 321 enumeration areas (EAs) (20 households were randomly selected from each urban EA, and 30 households were selected from each rural EA) in 13 out of 27 Indonesian provinces that were selected representing 83% of the Indonesian population in 1993, more details in [35]. At household level, several randomly selected household members were asked to provide detailed individual information. Being a member of a selected household (15 years and above) was defined as members that reside in the same dwelling and share food from the same cooking pot [35]. In the IFLS-5, 16,204 households and 29509 (excluding 617 who were pregnant) 18 years and older individuals were interviewed with complete anthropometric measurements. In the IFLS-5 the household response rate was 90.5% [35]. Although the survey is longitudinal, we restricted our analysis to the IFLS-5 cross-sectional survey for persons 18 years and older, being the most recent national survey available assessing anthropometric measures.

### 2.2. Measures

#### 2.2.1 Weight status

*Anthropometric measurements.* Heights were measured to the nearest millimetre using a Seca plastic height board (model 213) [35]. Weights were taken to the nearest tenth of a kilogram using a Camry model EB1003 scale [35]. Body mass index (BMI) was calculated as weight in kg divided by height in metre squared and classified according to Asian criteria: underweight (<18.5kg/m^2^), normal weight (18.5 to <23.0 kg/m^2^), overweight (23.0 to <25.0 kg/m^2^), obesity (≥25 kg/m^2^), class I obesity (25–29.9 kg/m^2^), and class II obesity (≥30 kg/m^2^) [36].

*Socio-demographic factor* questions included age, sex, marital status, education, work status, religion, residential status, subjective socioeconomic background, and country region. Subjective economic status was assessed with the question “Please imagine a six-step ladder where on the bottom (the first step), stand the poorest people, and on the highest step (the sixth step), stand the richest people. On which [economic] step are you today?” The answers ranged from (1) poorest to (6) richest [35].

*Childhood hunger* was assessed with the question “Did you experience hunger in your childhood (from birth to 15 years)?” [35] (Coded as yes).

*Fruit consumption* was assessed with three questions on, “How many days in the past week, (1) Banana, (2) Mango and (3) Papaya had been eaten?” [32]

*Food frequency consumption* was assessed with questions on, “How many days in the past week did you eat, 1a) Green leafy vegetables? (1b) carrots? (2a) banana? (2b) papaya? (2c) mango? (3) meat? (4) dairy products? (5) fish? (6) fast food? (7) fried snacks (*tempe, tahu, bakwan*, etc.)? (8) sweet snacks (*wajik, geplak*, donuts, wafers, chocolate, etc.)? and 9) soft drinks (Coca cola, sprite, etc.)?” [35] Inadequate fruit consumptions was defined as less than 3 days a week, and inadequate vegetable consumption less than daily. *Meal frequency* was measured with the question, “Do you normally eat, …3 times a day, 2 times a day, 1 times per day, 5–6 times a week, etc.?” [35] Responses were coded as 1 = 3 times/day and 0 = 2 times/day or less.

*Physical activity* was assessed with a modified version of the “International Physical Activity Questionnaire (IPAQ) short version, for the last 7 days (IPAQ-S7S)”. We used the instructions given in the IPAQ manual [37], and categorized physical activity according to the official IPAQ scoring protocol [38] as low, moderate and high.

*Tobacco use* was assessed with two questions: (1) “Have you ever chewed tobacco, smoked a pipe, smoked self-enrolled cigarettes, or smoked cigarettes/cigars?” (Yes, No) (2) “Do you still have the habit or have you totally quit?” (Still have, Quit) [35]. Responses were grouped into never, quitters and current tobacco users.

*Self-reported health status* was measured with the question, “In general, how is our health?” Response options were 1 = Very healthy, 2, Somewhat healthy, 3 = Somewhat unhealthy, and 4 = Unhealthy [35].

*Diabetes or high blood sugar, High blood pressure, High cholesterol (total or LDL)* were assessed with the question, “Has a doctor/paramedic/nurse/midwife ever told you that you had…?” (Yes, No) [35].

#### 2.2.2 Blood pressure (BP) measurements and classification

Three consecutive measurements of systolic and diastolic blood pressure (BP) were recorded with an Omron meter, HEM-7203, by regular trained interviewers on household members 15 years and older at home in a seated position [35]. Information was collected on awareness and treatment of hypertension. Average blood pressure was calculated arithmetically for the three measurements of each systolic and diastolic blood pressure. Blood pressure classification was done using JNC 7 algorithm [39]. Hypertension was defined as SBP ≥140 mm Hg and/or DBP ≥90 mm Hg and/or current use of antihypertensive medication. Normotension was defined as BP values <120/80 mm Hg in individuals who were not taking antihypertensive medication [39].

*Sleep disturbance* was assessed with five items from the “Patient-Reported Outcomes Measurement Information System (PROMIS)” sleep disturbance measure [40]. A sample item was, “I had difficulty falling a asleep.” Response options ranged from 1=not at all to 5= very much. (Cronbach's alpha = 0.68 in this study). Sleep disturbance was defined as a score of four or five on the averaged mean items.

The *Centres for Epidemiologic Studies Depression Scale* (*CES-D:* 10 items) was used to assess depressive symptoms, and scores 10 or more were classified as having depressive symptoms [41] (Cronbach's alpha = 0.71 in this study).

*Happiness* was assessed with the question, “Taken all things together how would you say things are these days – would you say you were very happy, happy, unhappy or very unhappy?” [35]

### 2.3. Data Analysis

Descriptive statistics were used to describe the variables. Multinomial logistic regression analysis was computed to calculate the adjusted relative risk ratios (ARRR) with 95% confidence interval (CI) to determine the associations between socio-demographic, health risk behavior, physical and mental health risk status variables and underweight and overweight or obesity. Potential multi-collinearity between variables was assessed with variance inflation factors, none of which exceeded critical value of 4. *P* < 0.05 was considered significant. Missing values were excluded from the analysis. “Cross-section analysis weights were applied to correct both for sample attrition from 1993 to 2014, and then to correct for the fact that the IFLS1 sample design included over-sampling in urban areas and off Java. The cross-section weights are matched to the 2014 Indonesian population, again in the 13 IFLS provinces, in order to make the attrition-adjusted IFLS sample representative of the 2014 Indonesian population in those provinces.” [35] Both the 95% confidence intervals and P-values were adjusted considering the survey design of the study. All analyses were done with STATA software version 13.0 (Stata Corporation, College Station, TX, USA).

## 3. Results

### 3.1. Sample Characteristics

The total sample included 29509 adults (median age 41.0 years, Inter Quartile Range (IQR) = 22.0, age range of 15–103 years) in Indonesia. The proportion of women was 52.1%, 55.8% had high school or higher education, 77.2% were married or cohabiting, 46.6% described themselves as having medium economic status and most (93.4%) belonged to Islamic religion. The majority of the participants (62.4%) worked for a salary, 52.0% were living in urban areas, and more than half (54.7%) lived in Java.

Overall, 11.2% measured to be underweight (13.5% among men and 9.1% among women), 39.8% normal weight (48.1% among men and 32.0% among women) and 49.0% had overweight or obesity (38.3% among men and 58.9% among women). Overall, 24.6% (CI = 24.1, 25.2) had class I obesity (25–29.9 kg/m^2^) and 8.5% (CI = 8.1, 8.8) had class II obesity (30 or more kg/m^2^). Among different age groups, underweight was the highest among 18–29 year-olds (16.7%) and those 70 years and older (29.8%), while overweight or obesity was the highest in the age group 30 to 59 years (more than 53%). The prevalence of underweight was significantly higher among male young adults (20.0%) than female 18–29 year-olds (13.2%), and in the age groups 30–49 years, while the prevalence of underweight in age groups 50 years and above was still higher among men than women but not significantly. Among the sexes, the prevalence of overweight or obesity was significantly higher in women than men in every age group (see Table 1).

Regarding various health variables, 8.8% of participants reported to have experienced hunger when they were a child, 57.7% consumed fruits less than three days a week, 59.9% did not consume vegetables daily, and 68.2% had meals three times a day. Other dietary behavior included, having 4–7 days a week meat (10.4%), 4–7 days a week dairy products (11.6%), 4–7 days a week fish (29.1), 4–7 days a week fried snacks (29.4), 4–7 days a week sweet snacks (17.1%), 4–7 days a week soft drinks (1.7%), and on one or more days a week fast food consumption (9.4%). Almost half (46.4%) engaged in low physical activity and 33.8% were current tobacco users. Almost one in five (18.9) rated their health as “very healthy”, 2.8% had diabetes, 33.5% hypertension, 4.9% high cholesterol (total or low-density lipoprotein), 3.0% sleep disturbance, 21.4% depression symptoms, and 11.8% rated themselves as being “very happy” (see Table 2).

**Table 1.** Sample and nutritional status prevalence by sociodemographic variables.

*Sociodemographic variables*	*Sample N (%)*	*Underweight (<18.5 kg/m^2^) %*	*Normal weight (18.5 to <23.0 kg/m^2^) %*	*Overweight/obesity (≥23 kg/m^2^) %*
**All**	29509	11.2 (10.8, 11.6)	39.8 (39.1, 40.4)	49.0 (48.4, 49.7)
Age in years				
18–29 (all)	8171 (22.7)	16.7 (15.8, 17.7)	49.0 (47.7, 50.2)	34.3 (33.1, 35.5)
18–29 (male)	3769 (24.9)	20.0 (18.6, 21.5)	53.6 (51.8, 55.4)	26.4 (24.9, 28.0)
18–29 (female)	4402 (20.7)	13.2 (12.1, 14.3)	43.8 (42.2, 45.5)	43.0 (41.4, 44.6)
30–39 (all)	8265 (22.5)	7.6 (7.0, 8.3)	38.6 (37.4, 39.8)	53.8 (52.6, 55.1)
30–39 (male)	4032 (21.8)	10.7 (9.7.11.8)	48.2 (46.4, 49.9)	41.1 (39.4, 42.9)
30–39 (female)	4233 (23.1)	4.9 (4.2, 5.7)	30.3 (28.7, 31.8)	64.9 (63.2, 66.5)
40–49 (all)	5622 (23.8)	5.6 (5.0, 6.3)	33.0 (31.7, 34.4)	61.4 (60.0, 62.8)
40–49 (male)	2792 (22.2)	7.5 (6.5, 8.7)	43.4 (41.4, 45.5)	49.0 (47.0, 51.1)
40–49 (female)	2830 (25.3)	4.1 (3.3, 4.9)	24.6 (22.9, 26.4)	71.3 (69.5, 73.1)
50–59 (all)	3866 (16.8)	8.6 (7.6, 9.6)	36.5 (34.8, 38.2)	54.9 (53.2, 56.6)
50–59 (male)	1773 (17.1)	8.8 (7.4, 10.4)	45.8 (43.3, 48.3)	45.4 (42.9, 47.9)
50–59 (female)	2093 (16.4)	8.4 (7.1, 9.8)	27.5 (25.5, 29.7)	64.1 (61.8, 66.3)
60–69 (all)	2120 (8.8)	14.7 (13.1, 16.5)	39.6 (37.3, 41.9)	45.7 (43.4, 48.1)
60–69 (male)	1006 (9.0)	15.9 (13.5, 18.5)	48.4 (45.0, 51.8)	35.8 (32.6, 39.1)
60–69 (female)	1114 (8.6)	13.6 (11.5, 16.0)	31.1 (28.2, 34.2)	55.3 (52.1, 58.5)
70 or more (all)	1465 (5.5)	29.8 (27.3, 32.5)	46.0 (43.2, 48.9)	24.2 (21.8, 26.7)
70 or more (male)	655 (5.0)	32.3 (28.4, 36.4)	49.4 (45.3, 53.6)	18.3 (15.4, 21.6)
70 or more (female)	810 (5.9)	27.9 (24.5, 31.5)	43.4 (39.6, 47.2)	28.7 (25.4, 32.3)
Sex				
Female	15482 (52.1)	9.1 (8.6, 9.6)	32.0 (31.2, 32.9)	58.9 (58.0, 59.8)
Male	14027 (47.9)	13.5 (12.9, 14.2)	48.1 (47.2, 49.1)	38.3 (37.4, 39.2)
Education				
None	1540 (6.4)	23.0 (20.7, 25.5)	44.2 (41.4, 47.0)	32.8 (30.2, 35.5)
Elementary	9671 (37.8)	11.2 (10.5, 11.9)	40.1 (39.1, 41.2)	48.7 (47.6, 49.8)
High school	14022 (43.7)	10.2 (9.7, 10.8)	40.2 (39.3, 41.1)	49.6 (48.7, 50.5)
Higher education	4221 (12.1)	8.5 (7.6, 9.5)	34.6 (33.0, 36.2)	56.9 (55.2, 58.6)
Marital status				
Unmarried	4123 (11.9)	21.3 (19.9, 22.7)	52.5 (50.8, 54.3)	26.1 (24.7, 27.7)
Married, cohabiting	22533 (77.2)	8.8 (8.4, 9.2)	38.1 (37.3, 38.8)	53.1 (52.6, 53.8)
Separated, divorced,	2853 (10.9)	17.2 (15.7, 18.8)	37.4 (35.5, 39.4)	45.4 (43.4, 47.4)
widowed				
Religion				
Islam	25378 (93.4)	10.8 (10.4, 11.2)	39.6 (38.9, 40.3)	49.6 (48.9, 50.3)
Other	2848 (6.6)	6.7 (5.7, 7.8)	38.9 (36.8, 40.9)	54.4 (52.3, 56.5)
Subjective economic background				
Poor	7156 (25.6)	12.6 (11.8, 13.5)	44.5 (43.2, 45.9)	42.8 (41.5, 44.1)
Medium	13269 (46.6)	10.0 (9.5, 10.6)	39.6 (38.6, 40.5)	50.4 (49.5, 51.4)
Rich	7809 (27.9)	9.4 (8.7, 10.2)	35.0 (33.8, 36.2)	55.6 (54.4, 56.9)
Work status				
Work for income	18399 (62.4)	10.7 (10.2, 11.2)	42.4 (41.5, 43.1)	47.0 (46.1, 47.8)
Search for job	365 (1.2)	22.6 (18.1, 27.8)	51.4 (45.6, 56.8)	26.1 (21.4, 31.3)
School attendance	822 (2.1)	25.0 (21.8, 28.5)	50.8 (46.3, 50.6)	24.2 (21.0, 27.7)
House keeping	8055 (27.5)	8.1 (7.5, 8.8)	31.8 (30.7, 32.9)	60.1 (58.9, 61.3)
Retired, sick, disabled,	1866 (6.8)	22.1 (20.3, 24.6)	42.6 (40.1, 45.1)	35.0 (32.6, 37.5)
other				
Residence	12177 (48.0)	12.7 (12.1, 13.4)	42.9 (41.9, 43.9)	44.4 (43.4, 45.4)
Rural	17332 (52.0)	9.8 (9.3, 10.3)	36.9, 36.1, 37.7)	53.3 (52.5, 54.2)
Urban				
Region				
Sumatra	6714 (22.8)	11.1 (10.8, 12.4)	40.8 (39.6, 42.1)	49.0 (47.8, 50.3)
Java	16137 (54.7)	11.2 (11.9, 12.9)	39.3 (38.5, 40.1)	49.4 (48.6, 50.3)
Main island groups^1^	6658 (22.6)	12.5 (11.7, 13.4)	41.2 (40.0, 42.5)	46.3 (43.4, 45.9)

Note: ^1^Major Island groups: Bali, West Nusa Tenggara, South Kalimantan, and South Sulawesi.

**Table 2.** Sample and BMI status by health risk behavior, physical and mental health status.

*Health variables*	*Sample N (%)*	*Underweight (<18.5 kg/m^2^) %*	*Normal weight (18.5 to <23.0 kg/m^2^) %*	*Overweight/obesity (≥23 kg/m^2^) %*
**Health risk behavior**				
Childhood hunger (≤15 years)	2412 (8.8)	10.6 (9.3, 12.1)	40.9 (38.7, 43.2)	48.5 (46.2, 50.8)
Fruit consumption (<3 days/week)	16462 (57.7)	11.4 (10.8, 11.9)	41.7 (40.8, 42.5)	47.0 (46.1, 47.9)
Vegetable consumption (<daily/week)	16263 (59.9)	11.1 (10.6, 11.6)	39.9 (39.0, 40.7)	49.0 (48.2, 49.9)
Meals a day (3 times vs 2 or 1 time)	19284 (68.2)	10.4 (9.9, 10.9)	40.8 (40.0, 41.8)	48.9 (48.0, 49.7)
Meat (4–7 days/week)	3115 (10.4)	8.9 (7.8, 10.1)	37.0 (35.1, 39.0)	54.1 (52.1, 56.1)
Dairy products (4–7 days/week	3469 (11.6)	11.9 (10.7, 13.1)	41.8 (40.0, 43.7)	46.3 (44.4, 48.2)
Fish (4–7 days/week)	9289 (29.1)	9.5 (8.8, 10.2)	39.4 (38.2, 40.6)	51.1 (49.9, 52.3)
Fast food consumption (1–7 days/week)	2982 (9.4)	9.6 (8.4, 10.8)	34.0 (32.1, 36.0)	56.4 (54.4, 58.4)
Fried snacks (4–7 days/week)	7236 (29.4)	9.5 (8.7, 10.2)	38.2 (37.0, 39.5)	52.3 (51.0, 53.6)
Sweet snacks (4–7 days/week)	5280 (17.1)	11.0 (10.0, 12.0)	39.6 (38.1, 41.1)	49.4 (47.8, 50.9)
Soft drinks (4–7 days/week)	574 (1.7)	12.1 (9.4, 15.3)	44.2 (39.5, 49.0)	43.7 (39.0, 48.5)
Physical activity				
Low	13591 (46.4)	10.6 (10.1, 11.2)	38.1 (37.1, 39.0)	51.3 (50.4, 52.3)
Medium	7483 (26.6)	9.7 (8.9, 10.4)	36.8 (35.5, 38.0)	53.6 (52.3, 54.9)
High	7160 (27.0)	11.2 (10.4, 12.1)	44.9 (43.6, 46.2)	43.9 (42.6, 45.2)
Tobacco use				
Never	18172 (61.1)	9.1 (8.6, 9.5)	33.2 (32.5, 34.0)	57.7 (56.9, 58.5)
Quit	1508 (5.2)	13.1 (11.2, 15.1)	38.3 (35.6, 41.1)	48.7 (45.8, 51.5)
Current	9829 (33.8)	14.8 (14.0, 15.6)	51.8 (50.7, 52.9)	33.4 (33.4, 34.5)
**Physical and mental health risk status**				
Self-rated health status	6581 (22.0)	12.8 (11.9, 13.8)	38.8 (37.5, 40.1)	48.4 (47.0, 49.8)
Unhealthy	17524 (59.1)	10.9 (10.3, 11.4)	40.2 (39.4, 41.0)	48.9 (48.1, 49.8)
Somewhat healthy	5404 (18.9)	10.4 (9.5, 11.4)	39.4 (38.0, 40.9)	50.1 (48.6, 51.7)
Very healthy				
Diabetes	704 (2.8)	3.7 (2.4, 5.5)	27.8 (24.3, 31.6)	68.5 (64.6, 72.2)
Hypertension	8807 (33.5)	8.2 (7.6, 8.9)	31.2 (30.1, 32.3)	60.5 (59.4, 61.7)
High Cholesterol (Total or LDL)	1310 (4.9)	1.9 (1.3, 2.9)	17.0 (14.9, 19.3)	81.1 (78.7, 83.3)
Sleep disturbance	913 (3.0)	13.3 (11.0, 16.0)	42.7 (39.1, 46.3)	44.0 (40.4, 47.7)
Depression symptoms	6420 (21.4)	11.9 (11.0, 12.8)	42.1 (40.7, 43.5)	46.0 (44.6, 47.4)
Happiness				
Unhappy	2499 (9.9)	13.8 (12.4, 15.4)	45.7 (43.5, 47.9)	40.5 (38.4, 42.7)
Somewhat happy	22065 (78.3)	10.4 (9.9, 10.8)	39.2 (38.4, 39.9)	50.5 (49.7, 51.2)
Very happy	3670 (11.8)	8.8 (7.8, 9.9)	37.0 (35.2, 38.8)	54.2 (52.4, 56.0)

Note: LDL= Low-density lipoprotein.

### 3.2. Associations of Underweight and Overweight or Obesity

Men were less likely to have overweight or obesity than women (Relative Risk Ratio: RRR = 0.67, Confidence Interval: CI = 0.61, 0.73). Compared with 18 to 29 year olds, 30 to 39 year-olds were less likely and individuals 70 years and older (RRR = 2.04, CI = 1.62, 2.56) were more likely to be underweight, and compared with 18 to 29 year olds 30 to 59 year-olds were more likely to have overweight or obesity and persons 70 years and older were less likely to have overweight or obesity (RRR = 0.53, CI = 0.43, 0.65). Individuals with high school or higher education were less likely underweight (RRR = 0.89, CI = 0.80, 0.99) and were more likely to have overweight or obesity (RRR = 1.28, CI = 1.19, 1.37). Persons with a richer subjective economic status (RRR = 1.35, CI = 1.23, 1.47) were more often having overweight or obesity than with persons from a poor subjective economic status. The odds of being underweight were higher among participants searching for a job (RRR = 1.63, CI = 1.20, 2.22), those attending school (RRR = 1.65, CI = 1.32, 2.05) and the retired, sick and disabled (RRR = 1.23, CI = 1.01, 1.49), and the odds of having overweight or obesity were lower in those searching for a job (RRR= 0.72, CI= 0.53, 0.96), those attending school (RRR = 0.53, CI = 0.43, 0.65) and higher among those who were house keeping (RRR = 1.15, CI = 1.06, 1.24) than those working for an income. Urban residence was negatively (RRR = 0.90, CI = 0.81, 0.99) associated with underweight and positively (RRR = 1.25, CI = 1.18, 1.34) associated with having overweight or obesity. Compared to living in Sumatra, living in the major island groups was positively associated with underweight (RRR = 1.18, CI = 1.04, 1.35) and negatively associated with having overweight or obesity (RRR = 0.83, CI = 0.76, 0.90).

Regarding various health risk behaviors, none of them were associated with underweight. Frequent meat consumption (RRR = 1.19, CI = 1.07, 1.31), frequent fried snacks consumption (RRR = 1.09, CI = 1.01, 1.17) and fast food consumption (RRR = 1.29, 1.16, 1.43) were positively, and having three meals a day (RRR = 0.82, 0.77, 0.88), frequent consumption of dairy products (RRR = 0.82, CI = 0.75, 0.90), frequent sweet snacks consumption (RRR = 0.90, CI = 0.83, 0.98) and infrequent fruit consumption (OR = 0.87, CI = 0.82, 0.93) were negatively associated with having overweight or obesity. In addition, high physical activity (RRR = 0.86, CI = 0.80, 0.93) and current tobacco use (RRR = 0.54, CI = 0.49, 0.59) were negatively associated with having overweight or obesity.

In relation to physical and mental health status, having diabetes, hypertension and high cholesterol were negatively associated with underweight and positively associated with having overweight or obesity. Very healthy perceived health status (RRR=1.12, CI=1.04, 1.22) and perceived very happiness (RRR = 1.19, CI = 1.09, 1.31) were both positively and depressive symptoms (RRR= 0.90, CI = 0.83, 0.97) negatively associated with having overweight or obesity (see Table 3).

**Table 3.** Multinomial regression predicting underweight and overweight or obesity.

	*Underweight ARRR*	*Overweight/obesity ARRR*
**Sociodemographics**		
Age in years		
18–29	1 (Reference)	1 (Reference)
30–39	0.62 (0.54, 0.70)***	1.92 (1.77, 2.09)***
40–49	0.55 (0.47, 0.65)***	2.27 (2.07, 2.49)***
50–59	0.79 (0.67, 0.94)**	1.65 (1.47, 1.84)***
60–69	1.27 (1.05, 1.55)*	1.09 (0.95, 1.26)
70 or more	2.04 (1.62, 2.56)***	0.53 (0.43, 0.65)***
Sex		
Female	1 (Reference)	1 (Reference)
Male	0.97 (0.84, 1.13)	0.67 (0.61, 0.73)***
Education		
None/Elementary	1 (Reference)	1 (Reference)
High school/Higher education	0.89 (0.80, 0.99)*	1.28 (1.19, 1.37)***
Subjective economic background		
Poor	1 (Reference)	1 (Reference)
Medium	0.92 (0.82, 1.03)	1.24 (1.15, 1.34)***
Rich	0.98 (0.86, 1.12)	1.35 (1.23, 1.47)***
Work status		
Work for income	1 (Reference)	1 (Reference)
Search for job	1.63 (1.20, 2.22)**	0.72 (0.53, 0.96)*
School attendance	1.65 (1.32, 2.05)***	0.53 (0.43, 0.65)***
House keeping	0.98 (0.86, 1.13)	1.15 (1.06, 1.24)***
Retired, sick, disabled, other	1.23 (1.01, 1.49)*	0.97 (0.83, 1.14)
Urban residence (base=rural)	0.90 (0.81, 0.99)*	1.25 (1.18, 1.34)***
Region		
Sumatra	1 (Reference)	1 (Reference)
Java	1.09 (0.97, 1.23)	0.91 (0.84, 0.98)*
Main island groups	1.18 (1.04, 1.35)*	0.83 (0.76, 0.90)***
**Health risk behavior**		
Childhood hunger (≤15 years) (base=no)	0.92 (0.77, 1.10)	1.07 (0.96, 1.20)
Fruit consumption (<3 days/week) (base ≥3 days/week)	1.04 (0.94, 1.15)	0.87 (0.82, 0.93)***
Vegetable consumption (<daily/week) (base=daily)	1.08 (0.98, 1.19)	1.02 (0.96, 1.09)
Meals a day (3 times) (base < 3 times)	0.92 (0.84, 1.02)	0.82 (0.77, 0.88)***
Meat (4–7 days/week) (base= <4 days/week)	0.88 (0.74, 1.03)	1.19 (1.07, 1.31)***
Dairy products (4–7 days/week) (base= <4 days/week)	1.08 (0.95, 1.24)	0.82 (0.75, 0.90)***
Fish (4–7 days/week) (base= <4 days/week)	0.91 (0.81, 1.02)	1.06 (0.98, 1.13)
Fast food consumption (1–7 days/week) (base=<1 day)	1.06 (0.90, 1.25)	1.29 (1.16, 1.43)***
Fried snacks (4–7 days/week) (base= <4 days/week)	0.94 (0.84, 1.06)	1.09 (1.01, 1.17)*
Sweet snacks (4–7 days/week) (base= <4 days/week)	1.07 (0.94, 1.21)	0.90 (0.83, 0.98)*
Soft drinks (4–7 days/week) (base= <4 days/week)	0.96 (0.71, 1.31)	1.06 (0.84, 1.34)
Physical activity		
Low	1 (Reference)	1 (Reference)
Medium	0.99 (0.89, 1.12)	1.01 (0.94, 1.09)
High	0.94 (0.84, 1.06)	0.86 (0.80, 0.93)***
Tobacco use (current)	1.12 (0.98, 1.28)	0.54 (0.49, 0.59)***
**Physical and mental health risk status**		
Self-rated health (very healthy) (base=not very healthy)	0.97 (0.86, 1.10)	1.12 (1.04, 1.22)**
Diabetes	0.53 (0.32, 0.87)*	1.29 (1.04, 1.60)*
Hypertension	0.67 (0.59, 0.76)***	2.13 (1.98, 2.30)***
High Cholesterol (Total or LDL)	0.49 (0.31, 0.79)**	2.67 (2.24, 3.19)***
Sleep disturbance	1.15 (0.90, 1.48)	0.86 (0.72, 1.02)
Depression symptoms	1.04 (0.93, 1.16)	0.90 (0.83, 0.97)**
Happiness (very happy) (base=not very happy)	0.90 (0.77, 1.04)	1.19 (1.09, 1.31)***

Note: ARRR = Adjusted Relative Risk Ratio; LDL = Low-density lipoprotein; *** *P* < 0.001; ** *P* < 0.01; * *P* < 0.05.

## 4. Discussion

The results of this national study demonstrate the co-existence of dual burden of underweight (11.2%) and overweight/obesity (≥23 kg/m^2^) (49.0%) and (≥25 kg/m^2^) 31.0% among 18 years and older individuals in 2014–2015 in Indonesia. These figures are similar to the 2013 national adult (18 years and older) prevalence of underweight of 11.1% and the prevalence of overweight or obesity (≥25 kg/m^2^) 30.4% in Indonesia [2],[3]. Largely similar rates of underweight and overweight or obesity have been reported in countries of the Southeast Asian region such as Bangladesh [5], Malaysia [6], Thailand [7] and Vietnam [8]. Further, the study showed a further decline in the prevalence of underweight from 14.4% in 2007 [14] to 11.2% in 2014–2015, and a further increase of overweight/obesity (≥25 kg/m^2^) from 17.9% in 2007 [14] to 31.0% (≥25 kg/m^2^) in 2014–2015 in Indonesia. This trend is consistent with a global decrease in the prevalence of underweight and increase of overweight or obesity over the past 20 years, which has also been observed in countries of the Southeast Asian region such as Vietnam [8] and Thailand [10].

The study found that the prevalence of underweight was the highest among 18 to 29 year-olds (16.8% overall, 20.0% in males and 13.2% in females) and those 70 years and older (29.8%). Previous studies also confirm the higher prevalence of underweight among young adults [14] and older adults [5],[14]. In this study the prevalence of underweight was in bivariate analysis significantly higher among men than women, while previous studies found an opposite result [8],[13],[14]. It is not clear why the prevalence of underweight is higher in men than women, partially also explained by a lack of data reporting undernutrition in adult men [5]. Reasons for the high prevalence of underweight during early adulthood may be related to food insecurity [4] and fear of being fat [16]. Some studies report an increase of an underweight body ideal and in eating disorders in Southeast Asia, including Indonesia [42]. In a small study among university students in Indonesia 14.5% of male and 5.1% of female students reported disordered eating attitudes.

In agreement with previous studies [5],[8],[13]–[15], this study found an association between lower education, not working and living in rural areas with underweight. Unlike some previous studies [4],[5],[14],[15], this study did not find an association between poorer economic background, the experience of childhood hunger and underweight. The association between participants attending school, retired, sick or disabled and underweight may be related to the higher prevalence of underweight among adolescents and older adults. Compared to persons living in Sumatra and Java, individuals living in major island groups (Bali, South Kalimantan, South Sulawesi and West Nusa Tenggara) had a higher prevalence of underweight. Hanandita and Tampubolon [14] found that undernutrition is spatially clustered within islands of Indonesia.

Regarding overweight or obesity, in consistence with previous studies [5],[8],[14],[15], this study found that being female, being middle aged, having higher education, higher economic status and urban residence were associated with having overweight or obesity. Compared to persons living in Sumatra, individuals living in Java major island groups had a lower prevalence of overweight or obesity. Hanandita and Tampubolon [14] found that over-nutrition is spatially clustered within islands of Indonesia. Further, specific dietary behavior (infrequent meals, frequent meat, fried snacks and fast food consumption) was, as previously found [18]–[22],[25],[26], associated with having overweight or obesity. It is likely that eating frequency impacts obesity through a combination of different mechanisms affecting a decrease in hunger, an increase of satiety responses and a reduction binge eating. Contrary to some studies [19],[23], this study found that inadequate fruit consumption and frequent sweet snacks consumption were negatively associated with having overweight or obesity. These findings largely confirm a shift in dietary behavior towards increased consumption of calorie-dense foods containing refined carbohydrates, fats, red meats, and low fiber in Indonesia.

In line with previous studies [6],[18],[19],[27],[28], this study also found a negative association between physical activity, smoking and having overweight or obesity. In this study, however, the association between physical inactivity and overweight or obesity was only observed in men and not in women (in sex-stratified analysis, not shown). Similar results were found in the Malaysian national study [6]. A possible explanation for this result could be that a greater proportion of men than women engage in high physical activity producing an effect large enough to reduce obesity [6].

The study findings further suggest, as previously found [3],[6],[29], that cardiometabolic comorbities, including diabetes, hypertension and dyslipidaemia, were associated with having overweight or obesity. Contrary to a previous study on self-rated health status [28], and in agreement with a previous study on happiness [34], this study found an association between very healthy self-rated health status, very happy happiness status and having overweight or obesity. Moreover, unlike previous studies on depression [30],[31], this study found a negative association between depression and having overweight or obesity. Some previous study [33] found an association between poor sleep quality and having obesity, while this study did not find such an association. Perhaps, overweight or obesity is further shaped by the ideal body image of fatness as a symbol of nurturance and affluence [14],[34]. Sohn [34] proposes that the positive relationship between having obesity and happiness may be “explained by improved socioeconomic status and health enjoyed by the obese”. In case Indonesians perceive overweight or obesity status as a signal of better health and greater happiness, it will be more difficult to target obesity in intervention programmes.

### Study limitations

The study was a cross-sectional study and the temporal relationships between sociodemographic factors, health status and health risk behaviors and underweight and overweight or obesity cannot be established in such studies; further longitudinal studies are needed. Apart from anthropometric and blood pressure measurements, a limitation of the study was that all the other information, including physical activity, was collected based on self-reporting.

## 5. Conclusion

The paper found in a cross-sectional study a high prevalence of both underweight and overweight or obesity among 18 years and older individuals in 2014–2015 in Indonesia, with underweight decreasing and overweight or obesity increasing in the past 20 years, as assessed in previous other studies. Sociodemographic, health risk behavior and health status risk factors were identified which can guide much needed public health interventions to address both these conditions.
